# Anterior talofibular ligament lesion is associated with increased flat foot deformity but does not affect correction by lateral calcaneal lengthening

**DOI:** 10.1186/s12891-019-2827-2

**Published:** 2019-10-27

**Authors:** Stephan H. Wirth, Arnd F. Viehöfer, Sarvpreet Singh, Stefan M. Zimmermann, Tobias Götschi, Dominic Rigling, Lukas Jud

**Affiliations:** 1 Department of Orthopedics, Balgrist University Hospital, University of Zurich, Forchstrasse 340, 8008 Zürich, Switzerland; 2 Institute of Biomechanics, ETH Zurich, Zurich, Switzerland

**Keywords:** Adult acquired flatfoot, Anterior talofibular ligament, Flatfoot correction, Lateral calcaneal lengthening

## Abstract

**Background:**

Several risk factors for adult acquired flatfoot deformity (AAFD) have been identified in literature. To this date, little attention has been paid to the lateral ligament complex and its influence on AAFD, although its anatomic course and anatomic studies suggest a restriction to flatfoot deformity. The aim of this study was to assess the influence of the anterior talofibular ligament (ATFL) on AAFD and on radiologic outcome following common operative correction by lateral calcaneal lengthening.

**Methods:**

We reviewed all patients that underwent lateral calcaneal lengthening for correction of AAFD between January 2008 and July 2018 at our clinic. Patients were grouped according to the preoperative MRI findings into those with an intact ATFL and those with an injured ATFL. Two independent readers assessed common radiographic flatfoot parameters on preoperative and postoperative radiographs.

**Results:**

Sixty-four flatfoot corrections in 63 patients were included, whereby the ATFL was intact in 29 cases, and in 35 cases the ligament was injured. An ATFL lesion was overall radiologically associated with increased flatfoot deformity with a statistically significant difference between the two groups for preoperative talometatarsal-angle (*p* = 0.002), talocalcaneal-angle (*p* = 0.000) and talonavicular uncoverage-angle (*p* = 0.005). No difference between the two groups could be observed regarding the success of operative correction or operative consistency after lateral calcaneal lengthening.

**Conclusion:**

The ATFL seems to influence the extent of AAFD. In patients undergoing lateral calcaneal lengthening, the integrity of the ligament seems not to influence the degree of correction or the consistency of the postoperative result.

## Background

Adult acquired flatfoot deformity (AAFD) is a commonly encountered problem in orthopaedic surgery, with an estimated prevalence of 3% [1]. Several risk factors of AAFD have been identified in literature including obesity, age, as well as rheumatic diseases [2–6]. The deformity is defined as a collapse of the medial longitudinal arch of the foot with a plantar and medial movement of the talar head at the level of the talonavicular joint, with consecutive forefoot abduction and increased eversion of the subtalar joint [3, 7]. One of the key structures in developing an AAFD is the tibialis posterior tendon, which typically shows attenuation or a tear in these patients [5, 8]. Several authors have paid attention to the medial ligamentous complex, and more specifically the deltoid ligament, as an insufficiency of this ligament complex allows the talus to tilt into a valgus position [9–11]. To this date however, little attention has been paid to the lateral ligament complex. In a cadaver study, Hintermann et al. found that transection of the anterior talofibular ligament (ATFL) results in a higher degree of calcaneal eversion [12].

Once conservative measures have failed, operative correction of AAFD may become necessary. Lateral calcaneal lengthening osteotomies, such as the Evans- or Hintermann-Osteotomy, are well-established operative treatment methods for correction of AAFD [13, 14]. By lengthening of the lateral column, the medial longitudinal arch is restored with induced adduction and supination of the forefoot [14, 15]. Both of these procedures have been shown to reliably produce satisfactory clinical results [15, 16].

When viewing the anatomical course of ATFL with its origin at the tip of the fibula and insertion on the lateral talus, it seems reasonable that the ATFL would restrict medial and plantar deviation of the talus and thus probably is one of the factors restricting AAFD. In lateral calcaneal lengthening, the bony correction results in a reduction movement of the talus. However, it is unknown whether an intact lateral ligament complex is required for restoration of a physiological position of the talus. The goal of the present study therefore was to investigate the influence of the ATFL on AAFD and on radiological outcome following operative correction by lateral calcaneal lengthening.

## Methods

A retrospective cohort study was conducted. All patients treated with lateral calcaneal lengthening (i.e. Hintermann-Osteotomy or Evans-Osteotomy) for stage II AAFD at our clinic from January 2008 to July 2018 were included. The study was approved by the local ethics committee (Zurich Cantonal Ethics Commission, KEK-ZH 2018–01083) and all patients gave their informed consent for their participation in and publication of this study.

In total, 64 operations were included, containing 42 Hintermann-Osteotomies and 22 Evans-Osteotomies in 41 females and 22 males. At time of surgery the average age was 45.4 ± 18.4 years and the average BMI was 27.5 ± 5.2 kg/m2. Mean FU was 16.5 ± 13.1 months. Only in 10 cases the minimum FU was solely 3 months, in the remaining 54 cases, minimum FU was at least 6 months.

The type of calcaneal lengthening correction (Hintermann- or Evans-Osteotomy) was chosen based on the preference of the treating surgeon. Due to the similarity of the procedures, both operative techniques were handled as equal. Exclusion criteria were additional bony procedures on the affected hind foot (i.e. arthrodesis or osteosynthesis), lack of preoperative MRI of the hind foot, lack of pre- or postoperative complete conventional radiographs (i.e. weight bearing lateral and dorsoplantar views) with a minimum follow-up (FU) of 3 months. All patients who had not given their written consent to participate in scientific research studies were excluded as well. Indication for the operative treatment was based on pain caused by flatfoot deformity with failed prior conservative treatment. In case of pathological findings of the tibialis posterior tendon on preoperative MRI, inspection of the tendon during the operative procedure was carried out. Additional transfer of the flexor digitorum longus (FDL) tendon was performed in case of severe tendon degeneration or tear [8, 17].

**Fig. 1:**
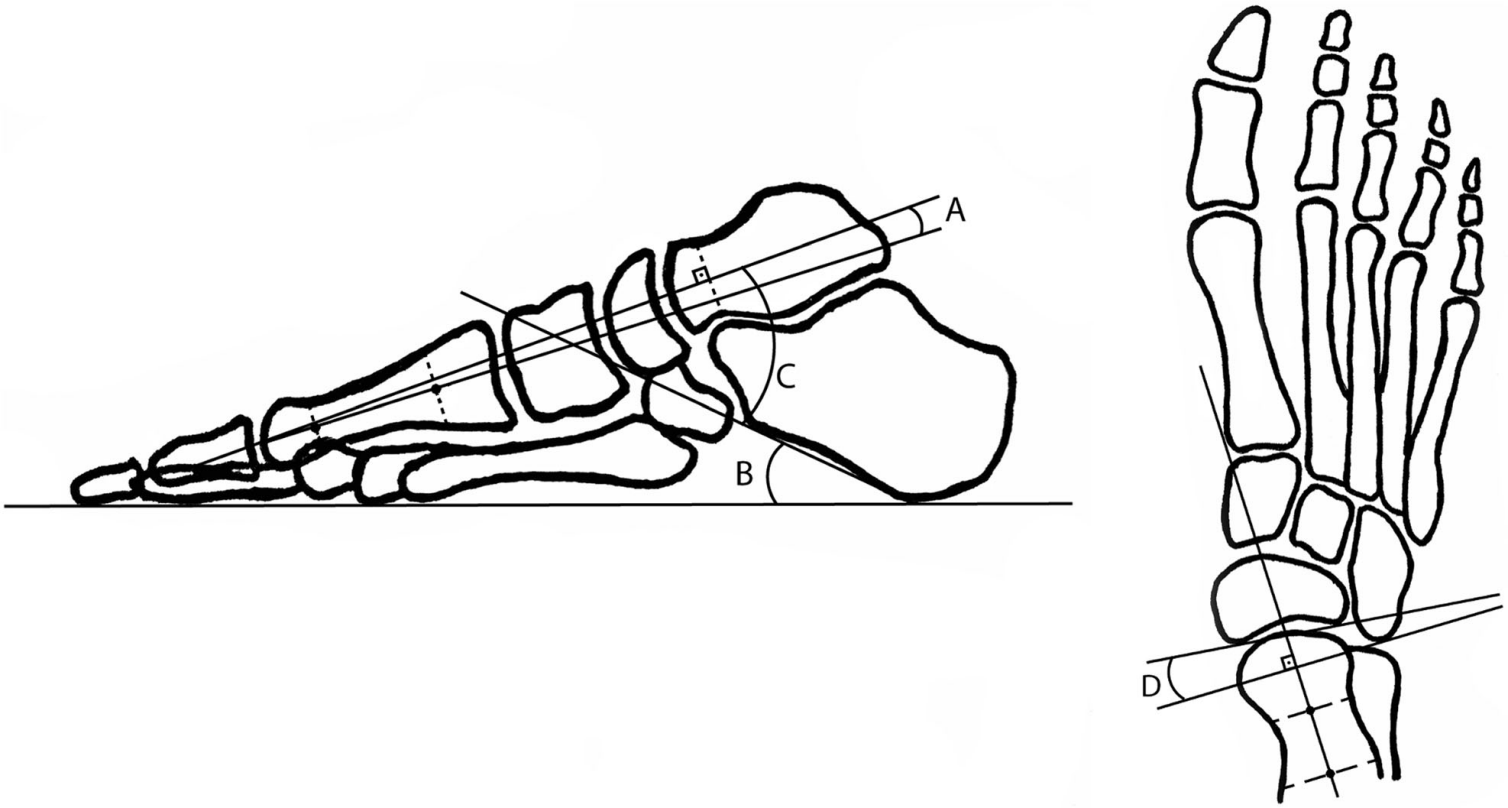
Talometatarsal-angle, **a** Calcaneal inclination-angle, **b** Talocalcaneal-angle, **c** Talonavicular uncoverage-angle, **d**

Preoperative data collection included BMI, condition of the ATFL and the deltoid ligament on MRI, and radiographic parameters commonly used to evaluate AAFD (talometatarsal-angle, calcaneal inclination-angle, talocalcaneal-angle, and talonavicular uncoverage-angle) (Fig. 1) [15, 18].

The ATFL and the deltoid ligament were assessed on preoperative MRI by two independent readers (JL, SS). Ligaments without any alterations were classified as intact, scarred or ruptured ligaments as injured. In case of a mismatch between the two readers, a trained foot-surgeon (VA) was consulted for further assessment in these cases. Subsequently, patients were divided according to the ATFL condition into two different groups: Group 1 with an intact ATFL; Group 2 with an injured ATFL.

Two independent readers (JL, SS) collected the radiographic parameters using conventional weight bearing lateral and dorsoplantar foot radiographs. As per definition, the talometatarsal-angle, talocalcaneal-angle, and talonavicular uncoverage-angle increase, and the calcaneal inclination-angle decreases with increasing flat foot deformity.

At 3 months FU and at last FU the same two readers collected the radiographic parameters in the same manner.

### Statistical analysis

Due to non-normal data distribution as well as unbalanced groups, non-parametric tests were employed. Differences between patients suffering from an injured ATFL and patients with an intact ligament were investigated using Mann-Whitney U testing. For each outcome, four parameters were derived:

- State at baseline (before surgery)
- Absolute change from baseline to 3 months FU
- Absolute change from baseline to last FU
- Absolute change from 3 months FU to last FU

Association of these four parameters was investigated using Mann-Whitney U tests. In order to additionally quantify the effect of a combined medial and lateral ligament injury of the preoperative primary outcomes of interest, the patient population was categorized into three categories which were defined as follows: (1) isolated lateral ligament injury, (2) combined lateral and medial ligament injury and (3) no lateral ligament injury. Median (range) angles using this categorization were tabulated and tested for statistical significance using a Kruskal Wallis test. Interrater reliability, concerning the radiographic parameters, was analyzed using intraclass correlation coefficients (ICC). Interclass correlation coefficients were interpreted according to Landis and Koch [19]. Referring the agreement between the two readers for ATFL assessment, agreement was tabulated and assessed using Cohen’s Kappa.

For further evaluation, mean values of both readers were used.

## Results

**Fig. 2:**
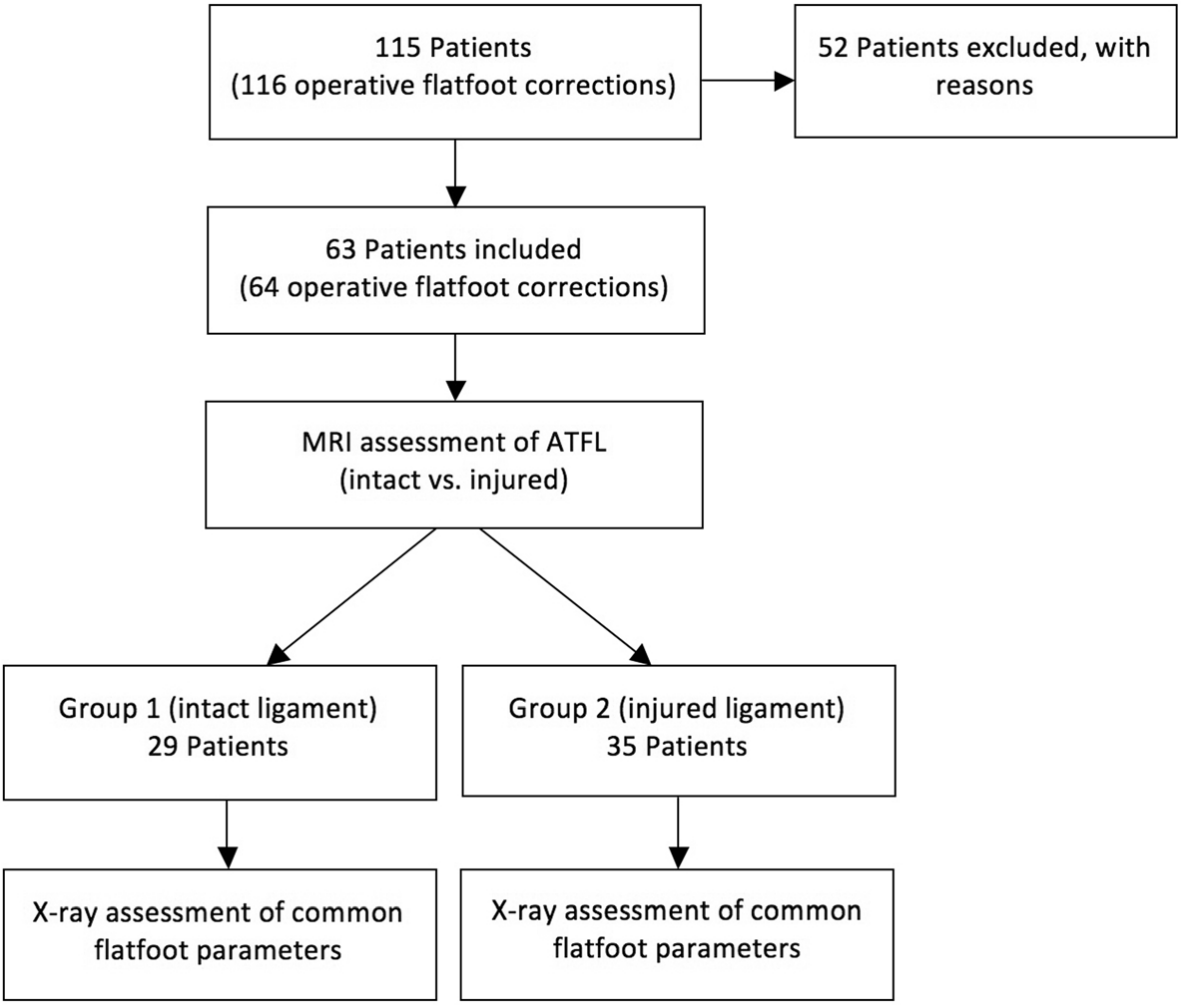
Flow-chart of inclusion and grouping of patients

Evaluation of the condition of ATFL on preoperative MRI revealed 29 intact ligaments (Group 1) and 35 injured ligaments (Group 2). Evaluation of the condition of the deltoid ligament revealed 40 intact ligaments and 24 injured ligaments. Overall, 20 patients showed a combined injury of the ATFL and the deltoid ligament. According to the patient grouping, Group 1 received a total of 8 Evans-Osteotomies and 21 Hintermann-Osteotomies, whereas Group 2 received 14 Evans-Osteotomies and 21 Hintermann-Osteotomies (Fig. 2).

An additional FDL transfer was necessary for 52% of the patients in Group 1 (15 of 29 cases), and in 66% in Group 2 (23 of 35 cases). Due to tendinopathy, debridement of the tibialis posterior tendon was performed in four patients in Group 1 and in three patients in Group 2. No statistical difference between the two groups was observed concerning the additional FDL-transfer or tibialis posterior tendon debridement (*p* = 0.496).

**Fig. 3:**
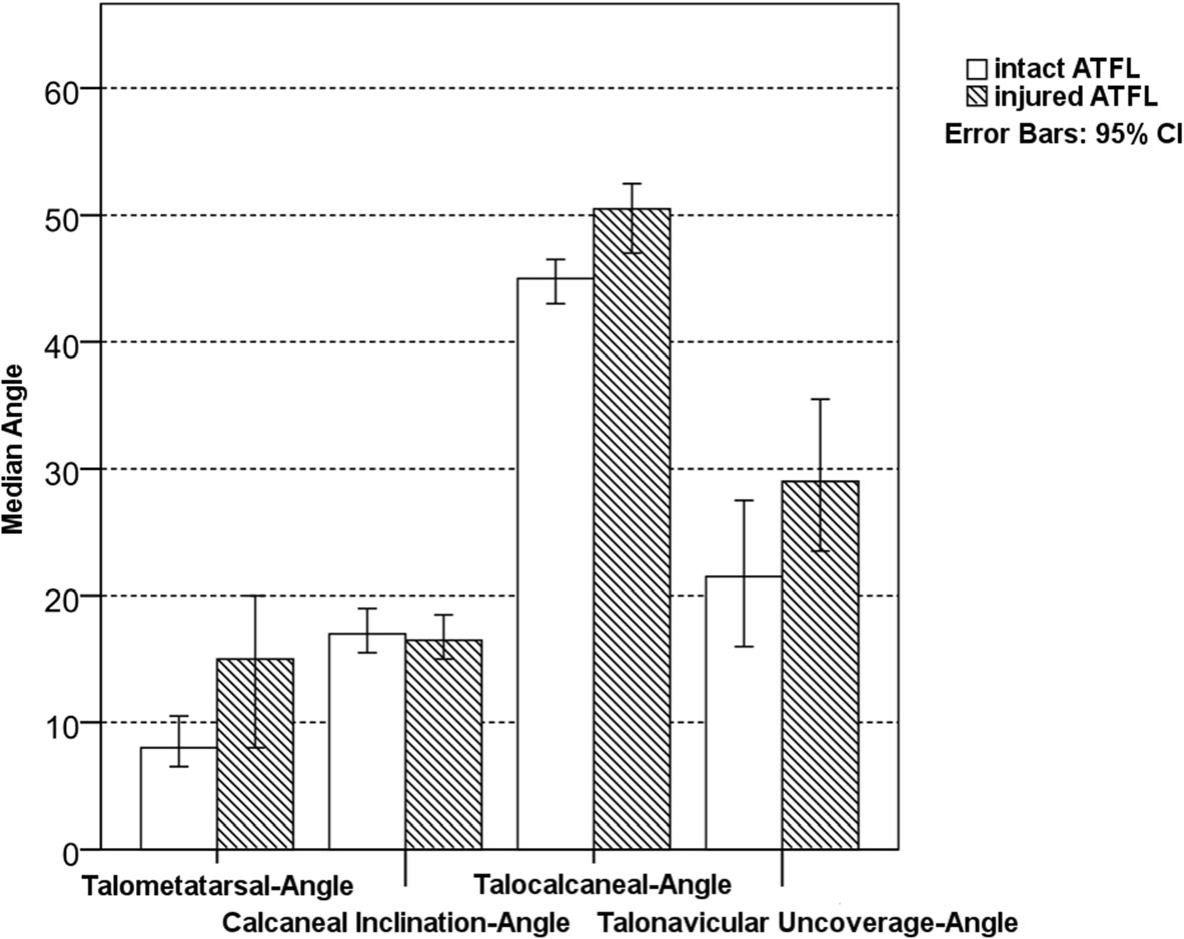
Preoperative mean values for measured radiographic parameters in Group 1 (intact ATFL) and 2 (injured ATFL)

**Fig. 4:**
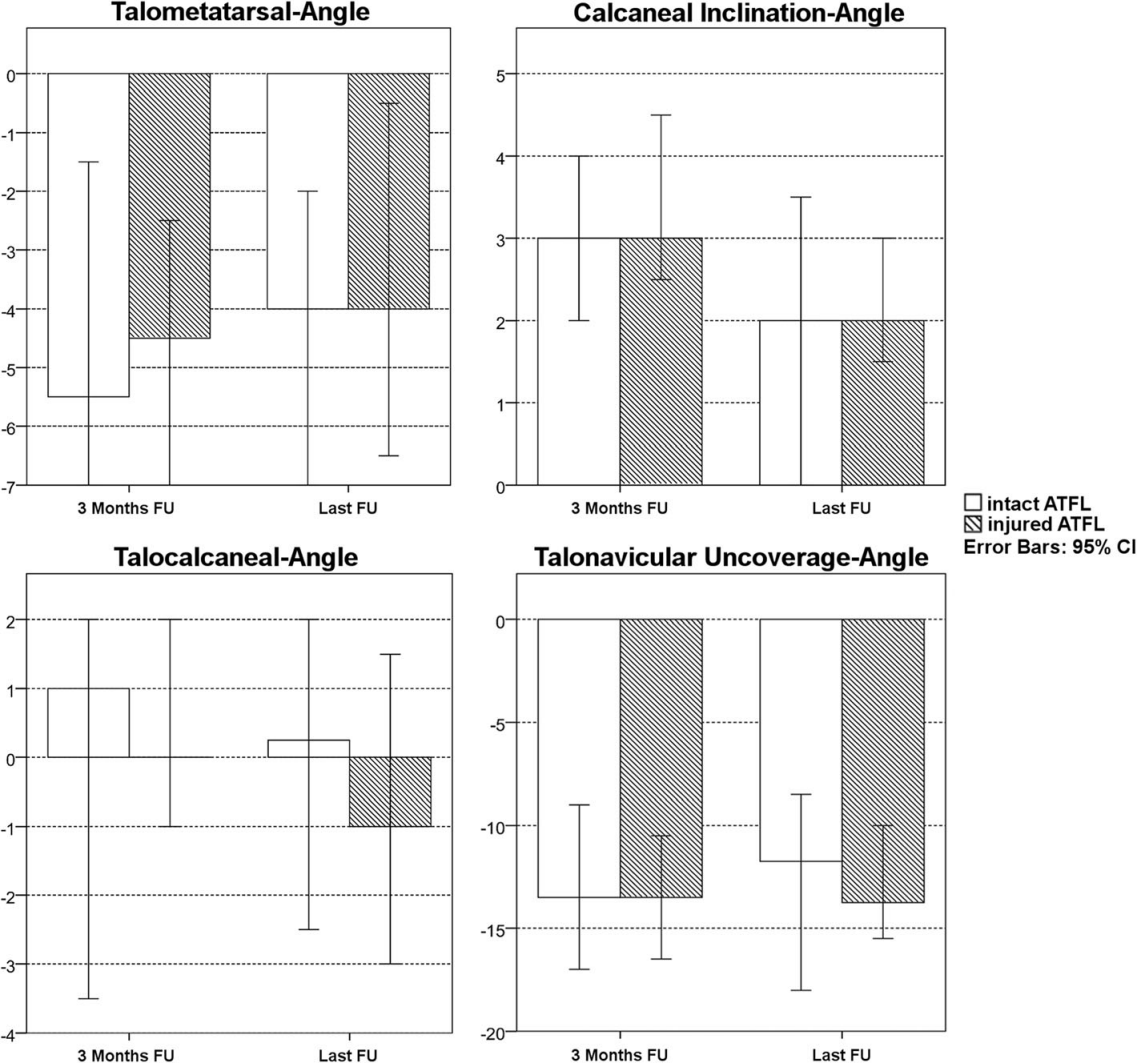
Postoperative changes in radiographic parameters for Group 1 (intact ATFL) and 2 (injured ATFL) from preoperative to each follow-up

Preoperative values for the radiographic parameters are shown in Table 1. A preoperative more severe AAFD could be observed in Group 2 (i.e. injured ATFL, Fig. 3), with statistically significant difference for talometatarsal-angle (*p* = 0.002), talocalcaneal-angle (*p* = 0.000) and talonavicular uncoverage-angle (*p* = 0.005). The calcaneal inclination-angle showed to be comparable between two groups without a statistical significant difference (*p* = 0.589) (Table 1). The degree of postoperative correction from preoperative to 3 months FU and last FU for both groups is illustrated in Fig. 4. Evaluation of these parameters showed no statistically significant difference between the two groups from preoperative to last FU (Table 2), neither from preoperative to 3 months FU (*p* = 0.602–0.803), nor from 3 months to last FU (*p* = 0.327–0.924). Additionally stratifying the population into isolated lateral ligament injury and combined medial and lateral ligament injury indicated medial ligament injury to have an additive effect on the observed associations. The model was significant for the outcomes of talometatarsal-angle (*p* = 0.002), talocalcaneal-angle (*p* = 0.001) and talonavicular uncoverage-angle (*p* = 0.020). No statistical significant difference was found for calcaneal inclination-angle (*p* = 0.861) (Table 3).

**Table 1:** Absolute values for preoperative radiographic parameters in Group 1 (intact ATFL) and 2 (injured ATFL)

	Group 1	Group 2
Talometatarsale-angle (°)		
Median	8.0	15.0
Min – max	0.5–15.0	1.0–32.0
*p*-Value	0.002	
Calcaneal inclination angle (°)		
Median	17.0	16.5
Min – max	10.0–25.0	7.5–26.5
*p*-Value	0.589	
Talocalcaneal-angle (°)		
Median	45.0	50.5
Min - max	30.0–59.0	38.5–64.5
*p*-Value	0.000	
Talonavicular uncoverage angle (°)		
Median	21.5	29.0
Min - max	−10.0 – 42.0	10.5–53.0
*p*-Value	0.005

**Table 2:** Absolute changes of radiographic parameters from preoperative to last FU in Group 1 (intact ATFL) and 2 (injured ATFL)

	Group 1	Group 2
Talometatarsale-angle (°)		
Median	−4.0	− 4.0
Min - max	−11.0 – 0.5	− 16.5 – 9.5
*p*-Value	0.829	
Calcaneal inclination angle (°)		
Median	2.0	2.0
Min - max	−1.5 – 7.0	−2.5 – 8.5
*p*-Value	0.493	
Talocalcaneal-angle (°)		
Median	0.25	−1.0
Min - max	−10.5 – 7.5	− 14.5 – 10.0
*p*-Value	0.544	
Talonavicular uncoverage angle (°)		
Median	−11.75	−13.75
Min - max	−25.5 – 6.5	−35.0 – 6.0
*p*-Value	0.603

**Table 3:** ATFL: anterior talofibular ligament; DL: deltoid ligament. Absolute values for preoperative radiographic parameters with additional stratified population into “isolated ATFL injury”, “combined ATFL and deltoid ligament injury”, and “No ATFL injury”

	Isolated ATFL injury (*n* = 15)	Combined ATFL – DL injury (*n* = 20)	No ATFL injury (*n* = 29)
Talometatarsale-angle (°)			
Median	12.5	19.0	8.0
Min - max	2.0–25.0	1.0–32.0	0.5–15.0
*p*-Value	0.002		
Calcaneal inclination-angle (°)			
Median	16.5	17.0	17.0
Min - max	8.5–23.5	7.5–26.5	10.0–25.0
*p*-Value	0.861		
Talocalcaneal-angle (°)			
Median	47.5	52.5	45.0
Min - max	38.5–53.0	38.5–64.5	30.0–59.0
*p*-Value	0.001		
Talonavicular uncoverage-angle (°)			
Median	25.0	31.5	21.5
Min - max	11.5–53.0	10.5–46.0	−10.0 – 42.0
*p*-Value	0.020		

The ICC between two readers, concerning the radiographic parameters, showed “almost perfect” agreement for the talometatarsal-angle (0.924), “substantial” agreement for the calcaneal inclination-angle (0.640), and “almost perfect” agreement for the talocalcaneal-angle (0.887) and talonavicular uncoverage-angle (0.948). Referring to the ATFL assessment, the two readers agreed in 50 cases and disagreed in 14 cases. This yields a moderate Kappa coefficient of agreement of k = 0.568 (*p* < 0.001).

## Discussion

To the best of our knowledge, the role of the ATFL in AAFD has not yet been investigated. In a cadaver study, Hintermann et al. were able to demonstrate that transection of the ATFL results in a higher degree of calcaneal eversion [12]. Based on this observation and its anatomical course, the ATFL might prevent the talus from rotating medially and thus restrict flatfoot deformity. There are several common radiographic flatfoot parameters describing the degree of AAFD [15, 18], and it is known that an increase of the talonavicular uncoverage-angle as well as the talometatarsal-angle are particularly associated with the onset of symptoms in flatfoot deformity [20]. In our study population, a significant difference in three out of four of the assessed established radiologic AAFD parameters could be observed, with a more pronounced deformity in patients with an injured ATFL. Accordingly, a positive correlation between AAFD and an injured ligament was found for these radiographic parameters. Only the calcaneal inclination-angle showed no statistically significant difference with comparable results between the two groups. Regarding the ICC between two readers it was also the calcaneal inclination-angle with just “substantial” agreement, the remaining parameters showed an “almost perfect” agreement, additionally underlining the positive radiologic correlation between AAFD and an injured ATFL. However, the ATFL seems to be just one of many factors, influencing the degree of AAFD. As it is already known that a deltoid ligament insufficiency predisposes the development of AAFD [9–11], we could also observe an additional accelerating effect on three out of four AAFD parameters in case of a deltoid ligament injury.

Concerning the postoperative situation, the degree of postoperative correction was the same whether the ATFL in MRI was intact or injured. Therefore, the hypothesis that an intact ATFL improves the amount of postoperative correction in lateral calcaneal lengthening in AAFD could not be confirmed. This might be due to the fact that the talus is reoriented laterally by bony correction, and soft tissue tensioning probably does not play a significant role during lateral calcaneal lengthening surgery. This hypothesis gets further reinforced by the observed relevant pressure changes after lateral calcaneal lengthening osteotomy in the calcaneocuboid joint, and therefore outside of the course of the ATFL [21–23]. Based on the findings of this study, it cannot be predicted whether additional tensioning of the ATFL in AAFD correction by lateral calcaneal lengthening would result in any additional benefit.

This study has some limitations. First of all, the condition of the ATFL was assessed on the preoperative MRI only. Clinical instability in patients with an injured ligament was not assessed due to lack of consistency of documentation in the retrospective analysis of the patient’s data. Vice versa, patients with an intact ligament on MRI were not assessed clinically for the same reason. Nevertheless, it is known that MRI shows high specificity and accuracy in diagnosis of an ATFL injury [24]. It is known that patients suffering from lateral ankle instability need surgical stabilization in solely 10 to 20% [25]. The remaining patients will achieve a stable ankle with a conservative treatment, even though the ATFL will still present as injured in MRI. A patient grouping by clinically assessed ankle instability would therefore probably underestimate the potential for a more pronounced AAFD in some patients referring to the findings presented this study. Finally, the present study cannot answer the question whether the more severe flatfoot deformity results in an ATFL lesion or if it is the other way around and an injured ATFL is the reason for the more severe deformity. To answer this question and to address the possible potential of lateral ligament repair in AAFD, further studies are necessary.

## Conclusion

The present study shows an increased flat foot deformity in case of injured ATFL. The integrity of the ATFL might therefore play an unrecognized role in the development of flat foot deformity. However, in lateral calcaneal lengthening, the integrity of the ligament does not affect correction of the flatfoot.

## Data Availability

Anonymized source data can be obtained from the corresponding author on reasonable request.

## Abbreviations

AAFD: Adult acquired flatfoot deformity
ATFL: Anterior talofibular ligament
FU: Follow-up
ICC: Intraclass correlation coefficients
